# Transforming Intraoperative Breast Cancer Diagnosis through D-FFOCT and AI Integration

**DOI:** 10.31557/APJCP.2025.26.11.3877

**Published:** 2025-11-21

**Authors:** Alireza Negahi, Hossein Negahban, Mehdi Khosravi-Mashizi, Amirhossein Rahmani, Hossein Neamatzadeh

**Affiliations:** 1 *Breast Health & Cancer Research Center, Iran University of Medical Sciences, Tehran, Iran. *; 2 *Department of Plastic Surgery, School of Medicine, Iranshahr University of Medical Sciences, Iranshahr, Iran. *; 3 *Hematology and Oncology Research Center, Shahid Sadoughi University of Medical Sciences, Yazd, Iran. *

**Keywords:** D-FFOCT, artificial intelligence, breast cancer, intraoperative diagnosis, machine learning

## Abstract

This editorial discusses the transformative potential of integrating dynamic full-field optical coherence tomography (D-FFOCT) with artificial intelligence (AI) in intraoperative breast cancer diagnosis. Traditional methods, such as frozen pathology, often face limitations in speed and accuracy, which can impact surgical outcomes. D-FFOCT, offering high-resolution, real-time imaging of tissue microstructures without ionizing radiation, presents a non-destructive alternative that maintains specimen integrity. Coupled with AI, particularly deep learning algorithms, this technology has demonstrated impressive diagnostic accuracy and speed, significantly reducing intraoperative margin evaluation time. Despite challenges in implementing these innovations, such as the need for high-quality datasets and addressing algorithmic bias, the integration of D-FFOCT and AI promises to enhance decision-making, alleviate the burden on pathologists, and improve patient outcomes. This approach not only aims to optimize breast cancer surgeries but also has broader implications for the diagnosis and treatment of other tumor types, highlighting the importance of ethical considerations and collaborative efforts in advancing clinical practice.

**Dear Editor,**

Intraoperative breast cancer diagnosis is crucial for effective surgeries, particularly in breast-conserving surgery (BCS), where the goal is to remove tumors while preserving as much healthy tissue as possible. Traditional methods, such as frozen pathology, can be slow, rely heavily on skilled pathologists, and may compromise the integrity of tissue samples. However, emerging technologies are transforming breast cancer diagnosis and treatment. One promising approach is the combination of dynamic full-field optical coherence tomography (D-FFOCT) with artificial intelligence (AI), which could significantly improve the speed and accuracy of margin assessments during surgery. This integration offers a substantial opportunity to enhance decision-making and surgical outcomes for breast cancer patients.

The landscape of breast cancer diagnosis and treatment is changing rapidly due to advancements in technology and a better understanding of the disease. Breast cancer, the most prevalent malignant tumor among women, presents unique challenges in surgical management, particularly in balancing effective tumor removal with the preservation of healthy tissue. While traditional methods like frozen pathology have their merits, they also come with limitations, including delays, dependence on skilled personnel, and potential degradation of specimens. In this context, integrating optical imaging technologies, particularly D-FFOCT with AI, presents a revolutionary opportunity to enhance intraoperative decision-making.

D-FFOCT is becoming a valuable tool for intraoperative imaging, offering high-resolution, real-time visualization of tissue microstructures without the use of ionizing radiation [1]. Unlike conventional methods that require extensive preparation and can produce tissue artifacts, D-FFOCT provides a non-destructive alternative that maintains the integrity of specimens for further analysis. This real-time visualization of cellular structures is essential for surgeons aiming for clear margins during BCS. The technology operates on the principles of optical coherence tomography, utilizing light to capture high-resolution images of tissue microstructures in real-time [2]. Implementation of D-FFOCT in clinical settings requires specialized equipment, including a light source, a beam splitter, and a high-resolution detector, along with training for surgical teams to interpret the images effectively [1, 2].

The combination of D-FFOCT and machine learning has shown promise in enhancing diagnostic accuracy and speed. Several studies have explored this integration for improved image analysis in breast cancer diagnosis [3]. For instance, one study focused on the automatic diagnosis and classification of breast surgical samples using both static and dynamic FF-OCT. This research compared manual interpretation with two automated strategies: feature engineering (FE) and convolutional neural networks (CNNs). The CNN-based approach achieved an impressive 90% classification accuracy, with 92% sensitivity and 85% specificity, highlighting the potential of AI to streamline the diagnostic process within a rapid timeframe of just 10 minutes. Another study applied D-FFOCT to fresh breast and lymph node biopsies in a rapid diagnosis clinic, revealing a sensitivity of 77% and specificity of 64% compared to conventional histopathology. These findings emphasize the importance of training AI algorithms with diagnostic imaging data, suggesting that deep learning could enhance outcomes further [4].

Research on FF-OCT and Dynamic Cell Imaging (DCI) for intraoperative examination reported high sensitivities for breast cancer diagnosis, with DCI achieving specificities of up to 95.1%. In a prospective cohort study by Zhang et al. (2024), D-FFOCT combined with deep learning was evaluated for rapid intraoperative cancer diagnosis in 182 patients. The deep learning model trained on 10,357 patches achieved remarkable results: 97.62% accuracy, 96.88% sensitivity, and 100% specificity. This model misclassified only one invasive ductal carcinoma (IDC), and the non-destructive D-FFOCT imaging process significantly reduced intraoperative margin evaluation time to approximately 3 minutes, compared to the traditional 30 minutes required for histological assessments. This capability is vital in clinical settings where timely decisions are essential for patient management [1].

Despite these advancements, it is important to acknowledge the challenges and limitations associated with implementing D-FFOCT and AI in clinical settings. Training AI models requires large, high-quality datasets, which can be difficult to obtain. To address this challenge, collaborative efforts between hospitals, research institutions, and technology developers are essential to curate diverse datasets that reflect various demographics and tumor types. Moreover, ongoing efforts to mitigate algorithmic bias include implementing fairness-aware algorithms and conducting regular audits of AI performance across different patient populations. Addressing these challenges will be crucial for the successful integration of these technologies into everyday clinical practice.

The innovative intraoperative diagnostic process utilizing D-FFOCT and AI offers numerous advantages, particularly in addressing the global shortage of pathologists by alleviating their workload. This technology minimizes the consumption of valuable tissue specimens, preserving them for further testing or research. Additionally, the rapid and accurate imaging capabilities of AI-assisted D-FFOCT enhance surgical outcomes by reducing the incidence of re-excision surgeries due to incomplete tumor removal. From the patient’s perspective, this integration can significantly improve the surgical experience by facilitating timely treatment decisions, thereby reducing anxiety and increasing overall satisfaction with care. Furthermore, the ability to preserve healthy tissue while ensuring complete tumor removal contributes to better cosmetic outcomes and enhances the quality of life for patients. Beyond these benefits, the D-FFOCT and AI integration can lead to reduced healthcare costs by improving intraoperative diagnostic accuracy and minimizing the need for follow-up surgeries. This reduction in re-excision surgeries translates to decreased operating room time, lower anesthesia costs, and shorter hospital stays, ultimately alleviating the financial burden on both patients and healthcare systems. The combination of cost-effectiveness, improved surgical outcomes, and heightened patient satisfaction highlights the transformative potential of D-FFOCT and AI in breast cancer management.

The integration of technology, particularly AI, in breast cancer diagnosis has far-reaching implications that extend to other tumor types, enhancing its clinical applicability. As personalized medicine becomes increasingly prevalent, the demand for accurate and swift diagnostics grows, making this adaptability crucial. However, the implementation of AI in clinical settings must consider ethical issues, including patient consent, data privacy, and the potential for algorithmic bias. The future of breast cancer diagnosis will depend not only on the creation of innovative tools but also on how effectively these resources are utilized to improve patient outcomes and quality of life. By adopting this transformative approach, we can develop more effective, efficient, and compassionate cancer treatment strategies that ultimately benefit both patients and healthcare providers.

In summary, the integration of D-FFOCT and AI marks a significant breakthrough in the intraoperative management of breast cancer. As advancements in these technologies continue, it is crucial to evaluate their broader implications for surgical practices and patient care. Future research should prioritize the enhancement of AI model training, the mitigation of potential biases, and the application of these technologies across various tumor types. Specific avenues for forthcoming studies may involve multi-center clinical trials aimed at validating the efficacy of the D-FFOCT and AI integration within diverse healthcare settings. Moreover, fostering collaborations between technology developers and clinical practitioners will be vital in facilitating the practical implementation of these innovations.
